# Pathological fractures as an adverse prognostic factor in chondrosarcoma: Results of a systematic review, meta-analysis and institutional case series

**DOI:** 10.1016/j.jbo.2025.100735

**Published:** 2025-12-06

**Authors:** Julian P. Maier, Ida Peiss, Felix Klingler, Nikos Karvouniaris, Kilian Reising, Hagen Schmal, Georg W. Herget

**Affiliations:** a Department of Orthopedics and Trauma Surgery, Medical Center - University of Freiburg, Germany; b Berta-Ottenstein-Program, Faculty of Medicine, University of Freiburg, Germany; c Faculty of Medicine, University of Freiburg, Germany; d Comprehensive Cancer Center Freiburg (CCCF), Medical Center - University of Freiburg, Germany; e Department of Orthopedic Surgery, University Hospital Odense, Denmark

**Keywords:** Chondrosarcoma, Pathological fracture, Prognosis, Survival analysis, Meta-analysis, Bone sarcoma

## Abstract

**Background:**

Pathological fractures are recognized as a potential, adverse prognostic factor in primary bone sarcomas. While some studies suggest higher recurrence rates and reduced survival, robust data specific to chondrosarcoma (CS) remain limited. This study evaluates the prognostic relevance of pathological fractures (PF) in CS patients.

**Methods:**

A systematic review and *meta*-analysis were conducted according to PRISMA guidelines. Eligible studies included patients with CS of the bone, with comparative groups based on fracture status and oncological outcomes. Study design, quality, and endpoints were assessed, and pooled *meta*-analysis was conducted. Additionally, clinical data from an institutional cohort was analyzed retrospectively.

**Results:**

Nine studies, with a total of 1,185 patients and 245 PF cases, met the inclusion criteria. Meta-analysis revealed that PF status was significantly associated with inferior survival at 1–5 years (pooled OR 0.40; 95 % CI 0.26–0.62; p < 0.0001). The increased risk of death was particularly evident in dedifferentiated CS (pooled HR 1.96; 95 % CI 1.46–2.63; p < 0.00001). Institutional data supported these findings, that PF was associated with worse overall survival (HR 3.90; 95 % CI 0.69–21.98; p = 0.12) and progression-free survival (HR 3.17; 95 % CI 0.58–17.36; p = 0.18), although significance was limited by sample size.

**Conclusions:**

Pathological fractures entail a significantly adverse prognosis in chondrosarcoma, particularly in high-grade and dedifferentiated subtypes and within the first 5 years of follow-up. Our institutional data corroborates *meta*-analysis findings. These results underscore the importance of personalized surgical management and intensive outcome monitoring for CS patients presenting with PF.

## Highlights

• Pathological fracture is an adverse prognostic factor in chondrosarcoma.
• Meta-analysis reveals higher mortality, especially in dedifferentiated chondrosarcoma.
• The prognostic impact is most evident in short- to mid-term follow-up.
• Pathological fractures should be considered in surgical care and outcome monitoring contexts.

## Graphical abstract

**Figure:**
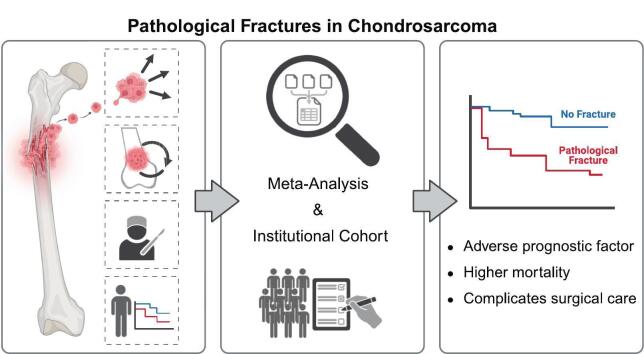

## 1 Introduction

Chondrosarcoma (CS) is the second most common primary bone sarcoma in adults [[1], [2], [3]]. Overall, CS accounts for ∼ 20 % of all malignant bone tumours. While ∼ 90 % of cases are classified as grade 1–3, dedifferentiated CS (ddCS) represents ∼ 10 %, alongside rare subtypes such as clear-cell and mesenchymal CS. [[4], [5], [6]]. The histological grades II and III, together with ddCS, are generally regarded as high-grade tumours [7,8]. Because CS reveals limited responsiveness to radio- and chemotherapy, surgical resection remains the mainstay and most effective treatment [2]. Reported 5-year survival rates vary substantially by histological grade, ranging from approximately 93 % for grade I, 74 % for grade II, 31 % for grade III, and as low as 18 % for ddCS [8,9].

Pathological fractures are present in 5–10 % of patients with a primary bone sarcoma overall, but in chondrosarcoma specifically, the reported rate may reach 25 % [[10], [11], [12]]. A lower extremity, particularly the femur, is the most commonly affected site [12]. Pathological fractures are often among the first complications at presentation, especially in case of high-grade malignant lesions [2].

In osteosarcoma, the presence of pathological fractures is well established as an adverse prognostic factor, associated with reduced overall survival and an increased risk of local recurrence [11,13]. In contrast, the prognostic relevance and clinical implications of pathological fractures in chondrosarcoma remain controversial [2,4,[14], [15], [16], [17]].

Therefore, the present study aims to review the literature on pathological fractures in CS systematically and to assess their impact on overall survival, as well as other clinically relevant outcome parameters (e.g., risk of local recurrence, risk of metastasis, adequacy of surgical margins). These findings are further correlated with data from our institutional tumor registry.

## 2 Methods

A systematic review was conducted in accordance with the Preferred Reporting Items for Systematic Reviews and Meta-Analyses (PRISMA) guidelines [18] and submitted to PROSPERO (ID: CRD420251057861), a database for registered systematic reviews and *meta*-analyses.

Clinical data from 31 patients diagnosed with primary chondrosarcoma treated at our Department of Orthopedics and Trauma Surgery, Medical Center - University of Freiburg, Germany, between 04/1997 and 07/2025, were included in this case series. Written informed consent was obtained from all patients, and all methods were conducted following the approved guidelines. Ethics approval was obtained from the local ethics committee of the University of Freiburg before the study commenced (protocol number 23-1206_3-S1).

### 2.1 Search strategy

**Fig. 1:**
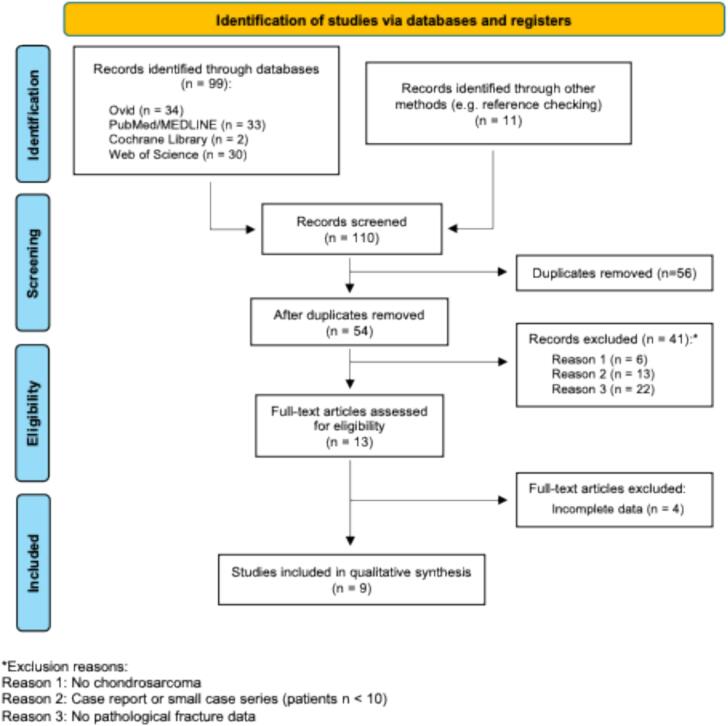
PRISMA flow diagram of study selection. Out of 110 identified records, 56 duplicates were removed. After screening and full-text assessment, 9 studies met the inclusion criteria and were included in our review. Reasons for exclusion are detailed at each stage. Figure visualized according to [18].

Relevant studies were extracted from Medline’s electronic databases (via PubMed, Ovid, and Web of Science) and the Cochrane Library. The systematic search strategy is shown in Fig. 1, with the final search term: (chondrosarcoma*) AND ((fracture*) OR (fractures*) OR (pathological fracture*) Fields] OR (pathologic fracture*)) AND ((recurrence*) OR (survival*) OR (overall survival*) OR (risk factor*) OR (prognosis*) OR (outcome*)) AND ((comparison*) OR (cohort*) OR (control*) OR (matched*)). Data was collected between 05/21/2025 and 06/20/2025.

### 2.2 Eligibility

Peer-reviewed articles published in English were included if they met the following criteria: studies reporting on patients diagnosed with chondrosarcoma of the bone, including a clearly defined group with a pathological fracture present at or before diagnosis or initiation of treatment, and a comparator group of patients without a pathological fracture. Studies had to report oncological outcome data (e.g., overall survival (OS), disease-specific survival (DSS), event-free survival (EFS), …). Eligible study designs included retrospective or prospective cohort studies, case-control studies, registry-based studies, or randomized controlled trials. Studies were excluded if they failed to provide information on the presence or absence of pathological fractures, lacked a comparator group, failed to report oncological outcomes, or were published before January 1, 2000. Additional exclusion criteria included case reports or small case series with fewer than 10 patients and review articles to avoid duplicating data.

Titles and abstracts were screened independently by two reviewers for eligibility based on the predefined inclusion and exclusion criteria. In cases where abstracts were unavailable, full-text articles were retrieved and assessed. Reference lists of included studies were manually screened to identify any additional eligible publications not captured by our initial search strategy. Patient demographics, number of patients, clinical outcome parameters, follow-up duration, and additional clinical data (e.g., surgical technique, recurrence rate, metastasis rate, …) were extracted whenever available.

We also retrospectively screened our institutional tumour registry to identify patients with a histologically confirmed diagnosis of chondrosarcoma treated at our institution. A total of 31 patients with chondrosarcoma were included in this case series. Patient demographics, tumour characteristics, treatment details, and outcome data including follow-up were extracted.

### 2.3 Endpoints and sub-analyses

The primary endpoint of our study was the overall survival of patients with chondrosarcoma with or without the presence of a pathological fracture. If OS data were not provided, EFS or DFS was used. Secondary endpoints included recurrence rate, risk of metastasis, or other clinically relevant parameters. The extent of reported data varied across studies, and not all parameters were provided consistently. To address this heterogeneity, we ran additional sub-analyses considering chondrosarcoma subtypes and the provided outcome parameters.

For our institutional cohort, overall survival (OS) was defined as the time from diagnosis to death, with survivors censored at the date of the latest follow-up. Progression-free survival (PFS) was defined as the time from surgery (or diagnosis if no surgery was performed) to the first event, defined as documented progression, recurrence, or death.

We hypothesized that pathological fractures in chondrosarcoma are associated with worse overall survival (OS) and inferior progression-free survival (PFS) compared to patients without a pathological fracture.

### 2.4 Statistical analysis

Data were collected and synthesized using Microsoft Excel (v16.94; Microsoft Corp., Redmond, WA, USA). Whenever available, outcome parameters were extracted directly from the included studies. If multiple hazard ratios (HRs) or Odds ratios (ORs) were reported, values from the final multivariate models were used. For studies providing survival data without explicit outcomes, survival status at fixed time points (e.g., 1-, 2-, and 5-year) was analyzed. Patients censored before a given time point were excluded from that analysis. HRs and ORs with 95 % confidence intervals (CIs) were calculated using standard formulas.

For studies not reporting numerical survival data, Kaplan-Meier (KM) curves were digitized using WebPlotDigitizer (v4.6; https://automeris.io/WebPlotDigitizer), as in previous studies [19,20]. Curves were calibrated to time (X-axis) and survival probability (Y-axis), and data points were extracted at predefined intervals. When exact values were unavailable, survival probabilities at target time points were estimated by linear interpolation between adjacent data points. Extracted survival rates, combined with reported group sizes, then served to calculate ORs and corresponding 95 % CIs.

Cox regression models were performed in R statistical software (v4.5.1; R Foundation for Statistical Computing, Vienna, Austria) using the *coxph()* function from the *survival* package, with pathological fracture included as a binary covariate. Kaplan-Meier curves were generated with *survfit*, visualized with *ggsurvplot* (*survminer* package), and group differences were tested using the log-rank test (*survdiff*). Overall survival (OS) was defined as the time from first presentation to death, with patients alive at last follow-up censored. Progression-free survival (PFS) was defined as the time from first surgery to first documented progression/recurrence or death, whichever occurred first. Patients without an event were censored at the last follow-up. Two-sided p-values < 0.05 were considered statistically significant.

For *meta*-analysis, a random-effects model with generic inverse variance weighting was applied to calculate pooled effect estimates using Review Manager (RevMan) (v5.4.1, Cochrane Centre, The Cochrane Collaboration, Denmark). Heterogeneity was assessed via Cochran’s Q and Higgins’ I2 statistics, with thresholds of < 25 %, 25–75 %, and > 75 % indicating low, moderate, and high heterogeneity, respectively. Statistical significance was set at p ≤ 0.10 for heterogeneity and p < 0.05 for pooled effects.

Comparisons of categorical variables, including the association between tumor localization (axial vs. appendicular), and the presence of pathological fractures or resection status (R0 vs. non-R0), were performed using Fisher’s exact test due to small sample sizes and expected cell counts below five. Survival outcomes (PFS, OS) were compared between axial and appendicular locations using the Mann–Whitney *U* test, as these variables were not normally distributed and sample sizes were unequal. All tests were two-sided, and a p-value < 0.05 was considered statistically significant.

### 2.5 Quality assessment

**Fig. 2:**
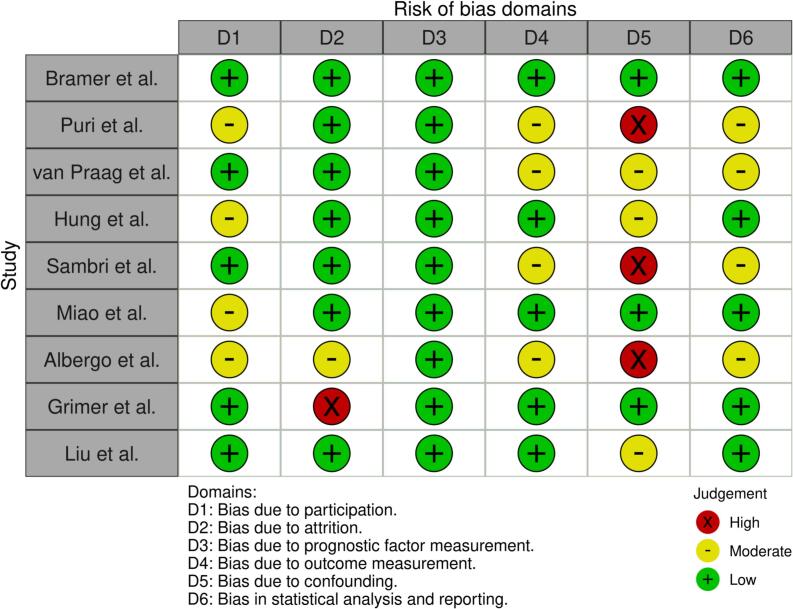
Risk of Bias assessment of included studies across six domains by using robvis and the QUIPS assessment tool for studies analyzing prognosis [39]. Low risk is depicted in green, moderate in yellow, and high in red. (For interpretation of the references to colour in this figure legend, the reader is referred to the web version of this article.)

The risk of bias was methodologically assessed by two independent authors, with disagreements resolved by consensus. Based on Cochrane’s recommendation, study quality was assessed using the QUIPS (Quality in Prognosis Studies) tool (Fig. 2) [21], which evaluates studies across six domains: study participation, study attrition, prognostic factor measurement (PF), outcome measurement, study confounding, statistical analysis, and reporting. Each domain is rated as low risk, moderate, or high risk.

## 3 Results

### 3.1 Study selection and characteristics

Our systematic literature search yielded a total of 99 records (Fig. 1). Another 11 were added through reference list checking. After removing duplicates (n = 56), 54 titles and abstracts were screened. Of these, 41 records were excluded according to our inclusion and exclusion criteria, leaving 13 articles for full-text review. Following a detailed assessment, a total of 9 studies met our predefined inclusion criteria and were included in the quantitative synthesis.

Study characteristics are summarized in the Table 1. The included studies were published between 2007 and 2024, comprising a total of 1.185 patients. Incidence of pathological fractures was 20.7 % (n = 245) compared to 79.3 % (n = 940) of patients without a fracture, respectively. All studies had a retrospective design, with cohorts ranging from 23 to 249 evaluable patients. Reported patient ages (mean or median, depending on the study) ranged from 45 to 66 years and the proportion of male patients from 46.3 % to 62.6 %. Tumour subtypes and grades varied between studies, with some focusing only on dedifferentiated chondrosarcoma (ddCS), while others also included chondrosarcoma grades 1–3 (CS G1-3). Follow-up times, reported as either median or mean, ranged from 13 to 113 months.

**Table 1:** Overview and characteristics of included studies summarizing study design, patient demographics, tumor features, follow-up, and outcome measures (R = retrospective, SC = single-center, MS = multi-center, OS = overall survival, HR = hazard ratio, OR = odds ratio, KM = Kaplan-Meier).

Study	Year	Country	Study Design	Patients (PF/Ctr)	Age	Sex (M/F in %)	Tumor Location	Tumor Grade (N)	Follow-up Duration (Months)	Exclusion Criteria	Outcome	Time point(s)	Effect Measure	Notes
Bramer et al.	2007	UK	R, SC	130 (33/97)	Median: 53–59	63.9/36.1	Upper and lower limb	G2 (82), G3 (17), DD (31)	Mean: 81 (3–263)	Metastasized; No surgical treatment; Unrelated Death	OS	10 y	OR	Reported directly
Puri et al.	2009	India	R, SC	45 (8/37)	Mean: 45 (15–76)	N/A	Axial and appendicular skeleton	G1 (11), G2 (23), G3 (11)	Mean: 43 (8–75)	Metastasized; Perioperative mortality; No follow-up	EFS	2 y	OR	Digitized KM curve
van Praag et al.	2024	Nether-lands	R, MC	249 (27/222)	Mean: 55 (±17)	55.4/44.6	Axial and appendicular skeleton	G2 (191), G3 (27), DD (31)	Minimum: 24 or prior event	Metastasized; tumor located above Th1 or phalanges.	OS (univariate)	2, 5, 10 y	HR, OR	Reported directly + Digitized KM Curve
Hung et al.	2023	USA	R, SC	67 (26/41)	Median: 61	61.2/38.8	Axial and appendicular skeleton	DD (67)	Mean: 21.5 (1–165.5)	N/A	OS (univariate)	2, 5 y	HR, OR	Raw data tables
Sambri et al.	2021	Italy	R, SC	175 (39/136)	Median: 66 (29–91)	46.3/53.7	Upper and lower limb	DD (175)	Median: 24 (3–275)	Non-surgical treatment; Pelvic or shoulder girdle location.	OS	2, 5, 10 y	OR	Digitized KM curve
Miao et al.	2019	USA	R, SC	72 (28/44)	Median: 60.5 (29–92)	62.5/37.5	Axial and appendicular skeleton	DD (72)	Median: 13 (1–227)	N/A	OS (multivariate)	−	HR	Reported directly
Albergo et al.	2015	UK	R, SC	182 (39/143)	Mean: 50 (8–90)	62.6/37.4	Femur	G1 (77), G2/3 (88), DD (17)	Mean: 113 (3–216)	Unresectable disease; palliative treatment.	DSS	5 y	OR	Survival proportions (Text)
Grimer et al.	2007	UK	R, MC	242 (41/201)	Median: 59 (15–89)	53/47	Axial and appendicular skeleton	DD (242)	N/A	Metastasized; palliative status.	OS (multivariate)	5 y	HR, OR	Reported + Survival proportions (Text)
Liu et al.	2017	China	R, SC	23 (4/19)	Mean: 50.4 (32–73)	52.2/47.8	Axial and appendicular skeleton	DD (23)	Mean: 16.57 (2–67)	N/A	OS (univariate)	1 y	HR, OR	Raw data tables

The most frequently assessed outcome parameters were overall survival (OS), disease-specific survival (DSS), and event-free survival (EFS). Effect measures included hazard ratios (HR) derived from uni- or multivariate Cox regression models and odds ratios (OR) calculated at fixed time points (1-, 2-, 5-, or 10-year survival). When not specifically reported, effect estimates were calculated from raw data tables, published survival proportions, or reconstructed from digitized Kaplan-Meier curves as described previously.

### 3.2 Risk of bias assessment

The overall risk of bias in most domains was mostly low. All studies clearly defined their study populations and outcome measures, resulting in a low to moderate risk of bias for the domains “study participation” (D1) and “outcome measurement” (D4). The moderate ratings mainly reflected the inclusion of heterogeneous chondrosarcoma grades (low to high grade) and limited adjustment for potential prognostic variables and confounders. Accordingly, several studies showed moderate to high risk of bias in the domain “confounding” (D5), particularly when only univariate analyses were performed or outcome parameters had to be reconstructed from digitized KM-curves. Moderate to high risk of bias was also observed in a few studies for “study attrition” (D2) due to insufficient information on follow-up data. Bias related to prognostic factor measurement (D3) and statistical analysis and reporting (D6) was generally rated as low to moderate. Taken together, despite some methodological limitations, most studies demonstrated acceptable internal validity and were considered suitable for quantitative synthesis.

### 3.3 Primary outcome

**Fig. 3:**
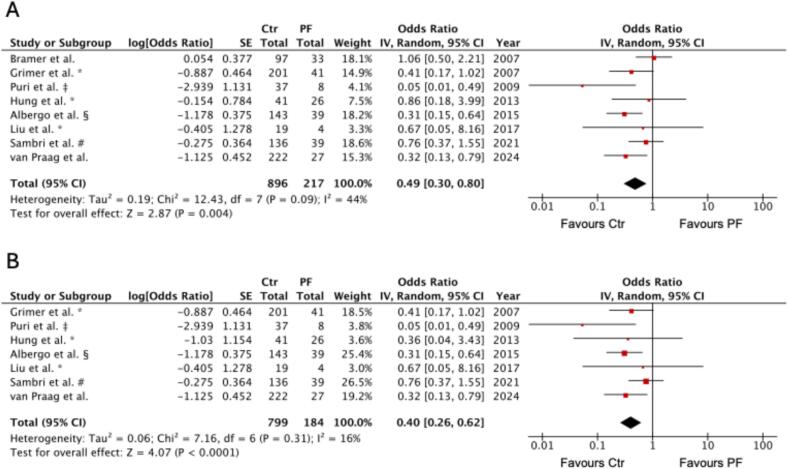
**Survival outcome in chondrosarcoma patients with versus without pathological fracture.** Forest plot of odds ratios (ORs) with 95 % confidence intervals (CIs) for survival outcome from 1- to 10-years (A), and 1- to 5-years follow-up (B). Squares represent study-specific ORs, with square size proportional to study weight; horizontal lines represent 95 % CIs; the diamond indicates the pooled effect size. ORs < 1 indicate a higher risk of death in the PF group (favours Ctr), whereas ORs > 1 indicate a higher risk of death in the control group (favours PF). Ctr, control group; PF, pathological fracture group. * only ddCS. # only ddCS of the limbs. § only CS of the femur and Disease-Specific-Survival (DSS) as outcome parameter. ‡ Event-Free-Survival (EFS) as outcome parameter.

**Fig. 4:**
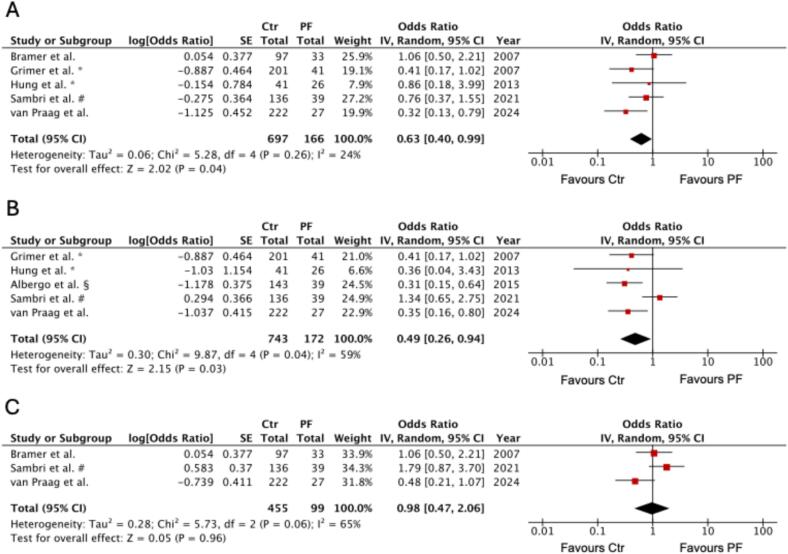
Forest plots of odds ratios (ORs) with 95 % confidence intervals (CIs) for overall survival (OS) from 1- to 10-years (A), 5-years (B), and 10-years (C) follow-up in CS patients with versus without pathological fracture. Pooled estimates were calculated using a random-effects model (generic inverse variance method) in RevMan v5.4.1. Squares represent study-specific ORs, with square size proportional to study weight; horizontal lines represent 95 % CIs; the diamond indicates the pooled effect size. ORs < 1 indicate a higher risk of death in the PF group (favours Ctr), whereas ORs > 1 indicate a higher risk of death in the control group (favours PF). *only ddCS subtype. # only ddCS of the limbs. § only CS of the femur and Disease-Specific-Survival (DSS) as outcome parameters.

A total of nine studies reported overall survival (OS) or related endpoints (disease-specific survival (DSS) or event-free survival (EFS)) with follow-up ranging from 1 to 10 years (Tab. 1). Of these, seven provided odds ratios (OR) that could be included in pooled analysis (Fig. 3A). Across these studies, the presence of a pathological fracture was associated with significantly worse survival (pooled OR 0.49, 95 % CI: 0.30–0.80, p = 0.006), indicating that patients without a pathological fracture were more likely to survive. Heterogeneity was moderate (I^2^ = 44 %, p = 0.09). We observed substantial variability in follow-up duration, outcome definitions, and histological subtypes (low to high grade and dedifferentiated chondrosarcoma); however, the overall direction of effect consistently favoured the non-fracture group. An additional sub-analysis (excluding studies only reporting DSS or EFS) also verified the favorable overall survival of patients without pathological fractures (pooled OR 0.64, 95 % CI: 0.43–0.95, p = 0.03; Fig. 4A).

This trend was particularly evident in the short-term sub-analysis that focused on 1- to 5-year follow-up periods. In this analysis, pathological fractures were consistently associated with reduced survival across the studies reviewed. The pooled odds ratio was 0.40 (95 % CI: 0.26–0.62, p < 0.0001), indicating markedly lower survival probabilities in patients with pathological fractures compared to those without them (Fig. 3B). Heterogeneity was low (I^2^ = 16 %, p = 0.31), suggesting that this effect was consistent, even with varying outcome definitions (OS, DSS, EFS) and diverse histological subtypes.

When restricting our analysis to a 5-year follow-up, patients with a pathological fracture demonstrated significantly poorer survival than those without a fracture (pooled OR 0.49, 95 % CI 0.26–0.94, *p* = 0.03; Fig. 4B). In contrast, no significant difference between groups was observed at the 10-year follow-up (pooled OR 0.98, 95 % CI 0.47–2.06, *p* = 0.96; Fig. 4C).

**Fig. 5:**
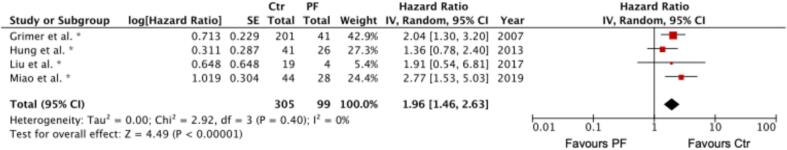
**Overall survival in dedifferentiated chondrosarcoma (ddCS) patients with versus without pathological fracture.** Forest plot of hazard ratios (HRs) with 95 % confidence intervals (CIs) for overall survival. Squares represent study-specific HRs, with square size proportional to study weight; horizontal lines represent 95 % CIs; the diamond indicates the pooled effect size. HRs < 1 indicate a higher risk of death in the control group (favours PF), whereas HRs > 1 indicate a higher risk of death in the PF group (favours Ctr). Ctr, control group; PF, pathological fracture group. *only ddCS.

In the subgroup analysis restricted to dedifferentiated chondrosarcoma (ddCS), a pathological fracture was associated with a nearly twofold increased risk of death (pooled HR 1.96, 95 % CI 1.46–2.63, *p* < 0.00001; Fig. 5). This finding concurs with the study by van Praag et al., which was the only study that provided individual hazard ratios for overall survival in a mixed cohort of CS G2/3 and ddCS, reporting a comparable HR of 2.22 (95 % CI: 1.26–3.91).

### 3.4 Secondary outcome

Secondary outcome parameters were reported across the included studies, with variable findings. Limb-salvage surgery was generally feasible in both groups, although Bramer et al. observed a slightly lower, but not significant, proportion thereof in patients with a pathological fracture (70 % vs. 81 %, *p* = 0.16) [12]. Adequate resection margins were achieved at similar rates in both fracture and control groups (51 % vs. 42 %, *p* = 0.31) in this study [12]. However, Miao et al. reported significantly fewer R0 resections among patients with a pathological fracture (54 % vs. 86 %, *p* = 0.010) [22], and Puri et al. reported inadequate margins in half of the fracture group [2].

Local recurrence rates revealed inconsistent patterns. Bramer et al. (33 % vs. 20 %, *p* = 0.11) and Albergo et al. (33 % vs. 26 %, n.s.) observed higher recurrence rates in the fracture group across all tumor grades, but identified a significant association only in patients with dedifferentiated chondrosarcoma [4,12]. In line with these results, Puri et al. also reported a higher rate of local recurrence in the fracture group [2]. By contrast, Sambri et al., Grimer et al., and Liu et al. reported no significant differences in recurrence rates [15,23,24]. Note that Sambri et al. demonstrated that amputation significantly reduced the risk of recurrence at 5 years, particularly in patients with pathological fractures [15]. With respect to metastasis, Grimer et al. found no association between fracture status and metastases at diagnosis, and Albergo et al. likewise failed to detect a higher risk of metastatic spread in fracture patients [4,23].

Only a few studies reported analyses examining the correlation between pathological fractures and anatomical localization. Grimer et al. identified axial location as an independent predictor of death (p = 0.016), with pathological fracture serving as an additional negative prognostic factor (p = 0.0019). [23] However, the presence of a pathological fracture did not increase the risk of inadequate surgical margins (26 % vs. 25 %, p = 0.55) in patients with dedifferentiated chondrosarcoma [23]. Bramer et al. reported that pathological fractures were associated with worse survival only in distally located tumours (p = 0.0268, univariate analysis), although a similar trend was observed across all subgroups [12].

### 3.5 Institutional case series

**Fig. 6:**
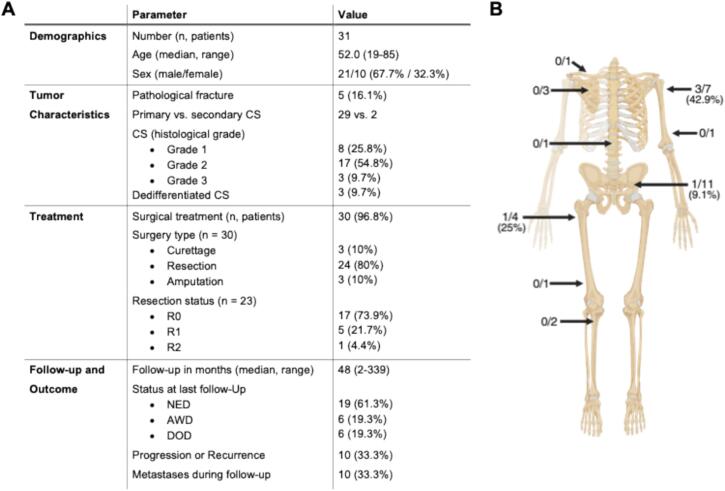
**Tumour distribution and clinical characteristics of patients with chondrosarcoma in the institutional cohort.** (A) Table summarizing patient demographics, tumor characteristics, treatment modalities, and outcomes. CS, chondrosarcoma; NED, no evidence of disease; AWD, alive with disease; DOD, died of disease. (B) Illustration showing the number of pathological fractures relative to the total number of tumours at specific sites. Values are presented as *x/*y, where *x* indicates the number of pathological fractures and *y* the total number of tumours at that site. Percentages are shown in parentheses. Parts of this figure were created in BioRender. Maier, J. (2025) https://BioRender.com/vodpxmv.

We analyzed 31 patients (Fig. 6) with a median age of 52 years (range, 19–85) and a slight male predominance of 67.7 %. Median follow-up was 48 months (range, 2–339). At the last follow-up, 19 patients presented no evidence of disease (NED), six were alive with disease (AWD), and six had died of disease (DOD). A pathological fracture at presentation was recorded in 16.1 % (n = 5) of patients. Two deaths occurred in the pathological fracture group (n = 2/5; 40 %) compared to four deaths in the control group without fracture (n = 4/26; 15.4 %). Surgery was performed in 96.8 % (30/31) of patients, with 80 % undergoing resection R0 resection was achieved in 73.9 %, with no difference between the fracture and control groups (75.0 % vs. 73.7 %).

**Fig. 7:**
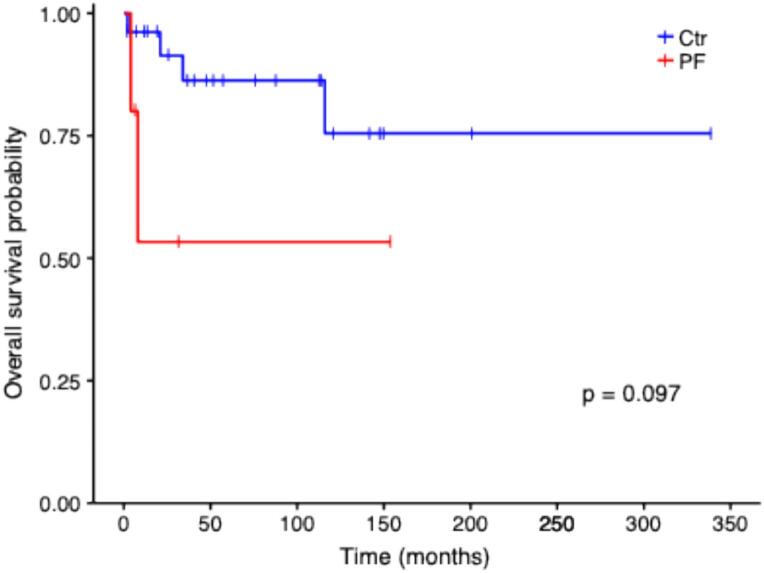
**Kaplan–Meier curve of overall survival by pathological fracture status in chondrosarcoma (n = 31).** Patients with pathological fracture (PF, red) showed poorer survival than those without fracture (Ctr, blue), particularly in the short- to mid-term follow-up, although the difference did not reach statistical significance (log-rank test, p = 0.097). Censoring is indicated by vertical ticks.

In univariable Cox regression, the presence of a pathological fracture was associated with an increased hazard of adverse outcomes for both overall survival (OS) and progression-free survival (PFS). The hazard ratio (HR) for OS) was 3.90 (95 % CI, 0.69–21.98; p = 0.12), and for PFS 3.17 (95 % CI, 0.58–17.36; p = 0.18). Although both estimates suggested a more than threefold increased risk, neither association reached statistical significance, likely due to the low number of events and small cohort size. Likewise, Kaplan-Meier analysis indicated a trend toward reduced survival in patients with a pathological fracture, particularly in the short- to mid-term follow-up, but the difference was not statistically significant in the log-rank test (p = 0.097) (Fig. 7).

When assessing anatomical distribution, pathological fractures occurred in 8.3 % (n = 1/12) of axial (e.g., pelvis, vertebrae) and 21.1 % (n = 4/19) of appendicular tumours, with no statistically significant difference between localizations (p = 0.62). Axial tumours showed a higher rate of inadequate margins, with R0 resections achieved in 50 % compared with 86.7 % in appendicular tumours, although this difference did not reach statistical significance (p = 0.131). Furthermore, no statistically significant association was observed between tumor localization (axial vs. appendicular) and progression-free survival (p = 0.584) or overall survival (p = 0.685) in our cohort.

## 4 Discussion

The present study aimed to assess the prognostic impact of pathological fractures on the overall survival (OS) of patients with chondrosarcoma. The adverse prognostic effect appears most evident during the short- to mid-term period (1–5 years), while the difference becomes attenuated over longer follow-up. Our time-stratified analyses indicate that this early survival disadvantage is most pronounced at 5 years in patients presenting with a pathological fracture, whereas no significant difference was observed at 10 years. This phenomenon probably reflects the selection of cohorts included, the majority of which consisted of ddCS − a subtype characterized by poor long-term outcomes, with reported survival rates as low as 10.5 % to 36.4 % irrespective of the patients’ initial presentation [9,15,25,26]. In this context, the presence of a pathological fracture may accelerate early disease progression or compromise local control, as suggested by higher rates of inadequate surgical margins, which could explain the survival gap within the first years [2,4]. In the long term, however, the ddCS prognosis is so dire that differences attributable solely to the fracture status seem attenuated. Notably, the concordance between our pooled analysis in ddCS and the independent results of van Praag et al. further supports the presence of a pathological fracture as a clinically relevant adverse prognostic factor even in this highly aggressive subtype [4,6,22,23]. Given the poor baseline prognosis of ddCS, our findings indicate that pathological fractures may accelerate disease progression further and warrant particular attention in both surgical decision-making and overall treatment planning.

Pathological fractures may have been hypothesized to promote the local dissemination of tumour cells through fracture hematomas, potentially compromising the ability to achieve adequate surgical margins [2,4,22]. Pathological fractures occur most frequently in high-grade tumours, particularly ddCS, where aggressive local destruction and rapid progression predispose to cortical breakthrough [4,26,27]. This predominance explains why most available data, including our *meta*-analysis, originate mainly from ddCS cohorts, while robust survival data on fracture cases in low-grade CS remain scarce. It is conceivable that the relative prognostic impact of fracture is even more substantial in low-grade CS, where the baseline prognosis is favorable. This hypothesis is supported by previous reports showing that pathological fractures adversely affected survival only in low-grade sarcomas [4]. In another large retrospective cohort of 341 patients with CS grades 1–3 and ddCS, Stevenson et al. identified pathological fractures as a significant predictor of disease-specific survival and local recurrence across all CS grades [16]. Our institutional data are consistent with these observations, although statistical significance was not reached, likely due to the limited number of events.

Available data on the risk of local recurrence or distant metastasis in patients with pathological fractures are inconsistent across the included studies, likely due to methodological rather than purely biological differences. Many cohorts were underpowered for subgroup or time‑dependent analyses, applied heterogeneous follow‑up schedules or surgical procedures, or did not stratify recurrence and metastasis by fracture status, thereby diluting any detectable associations. While local tumour cell dissemination through fracture-associated hematoma is a plausible contributing mechanism [2,4,22], it alone is unlikely to explain the variability observed across studies. Instead, it should be regarded as one of several interacting factors – including tumor burden, anatomical location, comorbidities, and the feasibility of achieving adequate surgical margins – that may collectively shape the prognostic impact of pathological fractures.

When interpreting these findings, one should consider the broader evidence on pathological fractures across other primary bone malignancies. In osteosarcoma, several large series and *meta*-analyses have consistently identified pathological fractures as an independent adverse prognostic factor, with lower 5‑year overall and event‑free survival [12,[28], [29], [30]], and an increased risk of developing metastasis [31]. However, no difference in recurrence rates was observed between osteosarcoma patients with or without a pathological fracture, and whether limb-salvage surgery or amputation was performed, provided that adequate surgical margins were achieved [12,31]. In contrast, Ewing sarcoma appears less strongly affected by the fracture status, with earlier studies reporting no significant difference in survival or local recurrence between patients with and without a pathological fracture [12,32,33]. For bone metastases, pathological fractures are typically regarded as a marker of advanced skeletal disease and poor performance status rather than an independent biological driver, and survival is determined mainly by the primary tumor and systemic disease burden [34]. In this context, our results indicate that the prognostic role of pathological fracture in chondrosarcoma resembles that of osteosarcoma more than Ewing sarcoma, which is generally considered more chemotherapy sensitive [35], or metastatic bone disease, but is strongly modulated by histologic subtype, particularly the dismal baseline prognosis of ddCS.

Different anatomical sites may influence not only chondrosarcoma biology but also the likelihood and clinical impact of pathological fractures. In our institutional cohort, pathological fractures were rare across all regions and showed no significant difference between axial (8.3 %) and appendicular (21.1 %) sites. Although axial tumours demonstrated numerically lower R0 resection rates and slightly shorter survival, these differences were not statistically significant, most likely due to the small cohort and number of events. Our findings are consistent with studies included in the review, which emphasize the prognostic relevance of tumor location, with axial or pelvic localization being linked to more difficult resections and poorer oncologic outcomes, including higher mortality [23,24]. However, most available studies reported only aggregated location data without patient-level linkage to fracture status, resection margins, or survival, preventing a pooled analysis. Overall, while our dataset did not show significant differences, the observed trends align with the broader literature, indicating that axial tumours – particularly those of the pelvis and spine – are known to pose greater surgical challenges due to proximity to vital structures, limited soft-tissue margins, and restricted resection options, ultimately carrying a higher risk of adverse oncologic outcomes [36,37].

On a final note, the type of pathological fracture may also play a critical role in surgical management and oncological outcomes. A non-displaced fissural fracture is likely to have different implications in terms of surgical complexity and the probability of achieving adequate resection margins than a displaced fracture accompanied by extensive hematomas [11,38]. Together, these findings underscore the need for future multi-institutional studies with standardized reporting of anatomical localization, margin status, and survival endpoints to disentangle the interplay among pathological fracture characteristics, tumor biology, and anatomical localization in chondrosarcoma.

## 5 Limitations

This *meta*-analysis has several limitations to be considered when interpreting our results. All included studies were retrospective, with inherent risks such as selection bias, incomplete data capture, and heterogeneity in treatment protocols. Differences in surgical strategies (e.g., limb salvage vs. amputation) and margin assessment across various centers further complicate direct comparisons. Outcome definitions and follow-up intervals also varied considerably, and most cohorts were comprised of primarily high-grade and dedifferentiated chondrosarcomas, limiting the generalizability of our findings to low-grade disease. Secondary outcome parameters could only be evaluated to the extent that the available data allowed, and were therefore unsuitable for pooled multivariate analyses. In our institutional cohort, multivariable adjustment for potential confounders (e.g., age, histologic subtype, tumor grade) was not feasible, as the events-per-variable were far below accepted thresholds and would have resulted in unstable estimates. Taken together, these limitations warrant caution, and the present results should be interpreted accordingly.

## 6 Conclusion

The presence of a pathological fracture constitutes a significant, adverse prognostic factor in chondrosarcoma, particularly in the short- to mid-term and in high-grade or ddCS. While its relative impact diminishes over longer follow-up, likely due to the uniformly poor prognosis of high-grade tumours, pathological fractures may also be an adverse determinant in low-grade disease, where survival is otherwise favorable. These findings underscore the need to integrate the pathological fracture status into the prognostic evaluation, surgical planning, and follow-up intervals. Further subtype-specific studies are required to validate these observations and to define optimal treatment strategies.

## CRediT authorship contribution statement

**Julian P. Maier:** Writing – review & editing, Writing – original draft, Visualization, Validation, Software, Project administration, Methodology, Investigation, Formal analysis, Data curation, Conceptualization. **Ida Peiss:** Writing – review & editing, Methodology, Investigation, Formal analysis, Data curation. **Felix Klingler:** Writing – review & editing, Conceptualization. **Nikos Karvouniaris:** Writing – review & editing. **Kilian Reising:** Writing – review & editing, Supervision, Resources. **Hagen Schmal:** Writing – review & editing, Supervision, Resources, Methodology, Conceptualization. **Georg W. Herget:** Writing – review & editing, Supervision, Resources, Project administration, Methodology, Conceptualization.

## Declaration of competing interest

The authors declare that they have no known competing financial interests or personal relationships that could have appeared to influence the work reported in this paper.
